# Identification of a prognostic classifier based on EMT-related lncRNAs and the function of LINC01138 in tumor progression for lung adenocarcinoma

**DOI:** 10.3389/fmolb.2022.976878

**Published:** 2022-08-17

**Authors:** Lingyan Xiao, Yongbiao Huang, Qian Li, Sheng Wang, Li Ma, Zhijie Fan, Zhe Tang, Xianglin Yuan, Bo Liu

**Affiliations:** ^1^ Department of Oncology, Tongji Hospital, Tongji Medical College, Huazhong University of Science and Technology, Wuhan, China; ^2^ Department of Pathophysiology, School of Basic Medicine, Tongji Medical College, Huazhong University of Science and Technology, Wuhan, China; ^3^ Department of Thoracic Surgery, Tongji Hospital, Tongji Medical College, Huazhong University of Science and Technology, Wuhan, China

**Keywords:** epithelial-mesenchymal transition, lncRNA, lung adenocarcinoma, prognosis, tumor microenvironment

## Abstract

**Purpose:** This study aimed to develop a prognostic indicator based on epithelial-mesenchymal transition (EMT)-related long noncoding RNAs (lncRNAs) and explore the function of EMT-related lncRNAs in malignant progression in lung adenocarcinoma (LUAD).

**Materials and methods:** A LUAD dataset was acquired from The Cancer Genome Atlas (TCGA) to identify prognostic EMT-related lncRNAs via differential expression analysis and univariate Cox regression analysis. Least Absolute Shrinkage and Selection Operator (LASSO) Cox regression analysis was utilized for variable selection and model construction. The EMT-related prognostic index (ERPI) was calculated according to the model and served as a classifier to divide LUAD individuals into high-ERPI and low-ERPI groups. A nomogram incorporating ERPI and clinicopathological variables was constructed. TCGA-LUAD, GSE50081, and GSE31210 were used to test the predictive capacity of the ERPI and nomogram. The characteristics of the tumor microenvironment (TME) were evaluated via the ESTIMATE, TIMER, and ssGSEA algorithms. Gene set variation analysis (GSVA) and ssGSEA were used to annotate the functions of the high-ERPI and low-ERPI groups. CCK8, transwell assay, wound-healing assay, and clone formation assay were conducted to clarify the biological functions of prognostic EMT-related lncRNAs.

**Results:** Ninety-seven differentially expressed EMT-related lncRNAs were identified, 15 of which were related to overall survival (OS). A prognostic signature was constructed based on 14 prognostic EMT-related lncRNAs to calculate the ERPI of each patient, and the predictive ability of ERPI was verified in TCGA, GSE50081, and GSE31210. The low-ERPI group survived longer and had a lower percentage of patients in advanced stage than the high-ERPI group. The nomogram had the highest predictive accuracy, followed by ERPI and stage. Patients with low ERPI had higher infiltration degree of immune cells and stronger immune responses than those with high ERPI. A series of *in vitro* experiments demonstrated that knockdown of *LINC01138* dampened variability, proliferation, and motility of A549 and H460 cells.

**Conclusion:** Our study developed a prognostic classifier with robust prognostic performance and clarified the biological functions of *LINC01138* in LUAD, aiding in making individual treatments for patients with LUAD and dissecting the mechanism of oncogenesis.

## Introduction

Lung cancer is not only the second most frequently diagnosed cancer but also the primary cause of cancer-related death worldwide, which accounts for 18% of cancer-related deaths (Sung et al., 2021). Although the survival of lung cancer has been extended in recent years due to advances in detection and treatment, only 22% of patients with lung cancer can survive 5 years or longer (Siegel et al., 2022). Lung cancer can be classified into diverse histological subtypes, and LUAD is the most common histological subtype, accounting for approximately 40% of lung cancer cases (Ganti et al., 2021).

EMT is known to play a fundamental role in embryogenesis, tissue regeneration, and malignant progression (Thiery et al., 2009; Pastushenko and Blanpain, 2019). EMT is characterized by the repressed expression of E-cadherin and increased expression of vimentin and can lead to changes in morphology, loss of cell polarity, reorganization of the cytoskeleton, and disassembly of cell-cell junctions. EMT is modulated by transcription factors, including the ZEB family, SNAIL, and TWIST1, which repress expression of epithelial markers and activate expression of mesenchymal markers. Interactions between TME and EMT are critical mechanisms in malignant progression. Constituents of TME can induce EMT by activating expression of EMT-related transcription factors or effector molecules, which in turn promote accumulation of immunosuppressive cells and expression of immunosuppressive molecules (Dongre and Weinberg, 2019). During the process of EMT, carcinoma cells enter a mesenchymal state and acquire malignant properties, including motility capacity, invasive behavior, metastasis, cancer stemness, and resistance to antitumor therapies (Pastushenko and Blanpain, 2019). A number of studies have underscored the significance of EMT in the development of lung cancer (Salazar et al., 2020; Yin et al., 2020; Deng et al., 2021).

Characterized as transcripts longer than 200 nucleotides, lncRNAs are important components of the transcriptome (Kopp and Mendell, 2018). Although lncRNAs are not translated into proteins, they regulate gene expression and control diverse cellular processes (Dangelmaier and Lal, 2020). Recent efforts have revealed the role of lncRNAs in tumorigenesis and malignant progression (Slack and Chinnaiyan, 2019; Wang et al., 2021a; Kim et al., 2021). Understanding the multiple functions of lncRNAs provides clues to dissect the mechanism of oncogenesis and aids in developing new strategies in cancer treatment. Many studies have indicated that lncRNAs can be potential prognostic predictors and therapeutic targets for patients with cancer (Wang et al., 2021a; Wu et al., 2021; Tang et al., 2022). The process of EMT can be regulated by lncRNAs such as *H19*, *MALAT1*, and *MEG3* (Yang et al., 2020a; Hu et al., 2020; Ji et al., 2020; Wang et al., 2020; Ye et al., 2020; Liu et al., 2021), and EMT-related lncRNAs have been demonstrated to be prognostic indicators in cancer (Tuo et al., 2018; Xu et al., 2020; Wang et al., 2021b). However, the prognostic potential of EMT-related lncRNAs in LUAD still remains to be explored.

The objective of this study was to identify prognostic EMT-related lncRNAs in LUAD and develop a classifier that can predict outcomes for patients with LUAD. The LUAD dataset in TCGA was acquired to identify prognostic EMT-related lncRNAs and construct a prognostic model, and three LUAD cohorts were used to validate the prognostic value of the model. *In vitro* experiments were conducted to explore the role of *LINC01138* in the development of lung cancer.

## Materials and methods

### Data acquisition and processing

Three LUAD cohorts, including the TCGA-LUAD dataset, GSE50081 and GSE31210, with transcriptomic data and clinicopathological information were acquired from the TCGA (https://gdc-portal.nci.nih.gov/) database and Gene Expression Omnibus (GEO) database (https://www.ncbi.nlm.nih.gov/geo/) (Okayama et al., 2012; Yamauchi et al., 2012; Der et al., 2014). The TCGA-LUAD dataset consists of 535 LUAD samples and 59 peritumoural normal samples. After those followed up for less than 30 days or without complete clinicopathological information were removed, a total of 822 LUAD samples were used in our study, including 469 samples from the TCGA-LUAD dataset, 127 samples from GSE50081, and 226 samples from GSE31210. The clinicopathological parameters of the above datasets are provided in Supplementary Table S1. A total of 1594 EMT-related genes were obtained from the Gene Ontology website (http://geneontology.org/), Epithelial-Mesenchymal Transition Gene Database (http://dbemt.bioinfo-minzhao.org/download.cgi), Molecular Signatures Database (https://www.gsea-msigdb.org/), GeneCard database (https://www.genecards.org/), and OMIM database (https://omim.org/). Pearson correlation analysis was used to determine the correlation between the expression of lncRNAs and EMT-related genes, and lncRNAs that met the threshold of correlation coefficient |r| > 0.3 and *p* < 0.001 were considered EMT-related lncRNAs. All the EMT-related genes and EMT-related lncRNAs are listed in Supplementary Tables S2, S3, respectively.

### Differential expression analysis

The expression of all samples in the TCGA cohort was normalized, and the genes with a mean expression less than 5 in all samples were discarded. Differential expression analysis was carried out using the “edgeR” package to identify all the differentially expressed genes (DEGs) based on the threshold of false discovery rate (FDR) < 0.05 and |log2-fold change (FC)| > 1 (Robinson et al., 2010; McCarthy et al., 2012; Chen et al., 2016). Next, the genes from DEGs, EMT-related lncRNAs, GSE50081, and GSE31210 were intersected by the “VennDiagram” package to obtain differentially expressed EMT-related lncRNAs. The “ggplot2” and “pheatmap” packages were used to create volcano plot and heatmaps, respectively, to present the differential expression of the EMT-related lncRNAs.

### Construction of an EMT-related lncRNA signature

The prognostic EMT-related lncRNAs were acquired from the differentially expressed EMT-related lncRNAs by univariate Cox regression analysis using the “survival” package. Lasso Cox regression analysis was executed using the “glmnet” package to screen prognostic EMT-related lncRNAs for model construction (Tibshirani, 1996; Friedman et al., 2010). A prognostic EMT-related model was constructed in the TCGA cohort based on candidate EMT-related lncRNAs and corresponding coefficients obtained in Lasso Cox regression analysis. Each patient with LUAD could obtain a score according to the prognostic model, which was named EMT-related prognostic index (ERPI). LUAD individuals in TCGA-LUAD, GSE50081, and GSE31210 were classified into high-ERPI and low-ERPI groups according to the median ERPI.

### Evaluation of the prognostic performance of ERPI

The predictive capacity of ERPI was validated in the TCGA cohort, GSE50081 and GSE31210. Principal component analysis (PCA) was utilized to confirm whether the high-ERPI and low-ERPI groups could be separated based on the prognostic EMT-related lncRNAs. Differences in the overall survival rate between the high-ERPI and low-ERPI groups were evaluated by Kaplan–Meier survival curves. The effect of the ERPI on survival was delineated via univariate and multivariate Cox regression analyses. The “survival” package was utilized to conduct the above survival analyses. (Therneau et al., 2000). The proportion of survivors in the high-ERPI and low-ERPI groups was calculated, and the ERPI between the surviving and nonsurviving patients was compared. The time-dependent receiver operating characteristic (ROC) curves were created using the “timeROC” package to show the predictive capacity of ERPI (Blanche et al., 2013). A nomogram based on the ERPI and clinicopathological variables was established to predict the survival probability of patients with LUAD. The C-index and area under the curve (AUC) were calculated to assess the predictive accuracy of the nomogram.

### Evaluation of the association between ERPI and clinicopathological parameters

The distribution of clinicopathological subtypes between the high- and low-ERPI groups was compared using the chi-square test, which is presented in the form of a heatmap. Then, patients were divided into diverse subgroups according to the clinicopathological characteristics, and the ERPI between these subgroups was compared by using the Wilcoxon test.

### Assessment of the TME characteristics in the two ERPI groups

A number of studies have reported interactions between EMT and the TME. Thus, we evaluated the relationship between TME characteristics and ERPI. The ESTIMATE algorithm was utilized to calculate the stromal score, immune score, and tumor purity, which represent the infiltration degree of stromal cells, immune cells, and tumor cells, respectively (Yoshihara et al., 2013). The infiltration degree of the major subtypes of immune cells in the TME was acquired via the TIMER and ssGSEA algorithms (Subramanian et al., 2005; Hänzelmann et al., 2013; Li et al., 2020). ssGSEA was also used to identify the related immune functions of the high- and low-ERPI groups. A study conducted a comprehensive analysis in 33 cancer types in the TCGA database to identify six immune subtypes associated with the immune environment and survival of patients with cancer (Thorsson et al., 2018). We assessed the link between the ERPI and the immune subtypes via the chi-square test, ggalluvial, and Kaplan–Meier survival curves.

### GSVA

GSVA was applied to identify the pathways and hallmarks that are associated with the ERPI by using the “GSVA” package (Hänzelmann et al., 2013). Enrichment scores were compared between the ERPI groups via the “Limma” package (Ritchie et al., 2015; Phipson et al., 2016). The pathways and hallmarks meeting the screening criterion of FDR <0.05 were considered to be differentially enriched between the two ERPI groups.

### Cell culture and transfection

The lung cancer cell lines A549 and H460 were purchased from the China Center for Type Culture Collection and cultured in RPMI-1640 medium (HyClone, United States) with 10% FBS (Gibco, United States) at 37°C with 5% CO^2^.

The siRNA of LINC01138 was transfected into lung cancer cells using Lipofectamine 3,000 (Invitrogen, United States). The siRNA target sequence for LINC01138 was as follows: 5′-CCU​CCU​CUU​CAG​CCU​ACU​U-3′.

### qRT-PCR

Total cell RNA was extracted using an RNA extraction kit (TaKaRa, Japan) and reverse transcribed using Hi Script II QRT SuperMix (Vazyme, China). Next, the qRT-PCR was run with a Real-Time PCR System (7900HT, Applied Biosystems, United States). The primers used in this study were as follows: LINC01138, 5′-TAT​TTA​CGA​AAG​CTG​AAA​GCG-3' (forward) and 5′-CTG​CAT​GGG​ATA​GGA​GAA​AC-3' (reverse); GAPDH, 5′-GAC​AGT​CAG​CCG​CAT​CTT​CT-3' (forward) and 5′-GCG​CCC​AAT​ACG​ACC​AAA​TC-3' (reverse) (Zhang et al., 2018).

### CCK8 assay

A total of 3,000 cells/well were seeded into 96-well plates overnight, and then the medium with 10% CCK8 (MedChem Express, United States) was incubated in each well for 1–2 h at different time points. OD values (wavelength of 450 nm) were detected by a microplate reader (BioTek, Winooski, VT, United States) to evaluate cell viability.

### Clone formation assay

A total of 1,000 cells/well were seeded in 6-well plates. After approximately 2 weeks, cell clones were fixed with methanol, stained with 1% crystal violet and photographed.

### Wound healing assay

Cells were seeded in 12-well plates, cultured for 12 h to 100% density, and then scratched with 10 μL pipette tips to create wounds. At 0 h and 48 h, images of wounds were captured, and the wound healing area was calculated using ImageJ software.

### Transwell assay

A total of 2 × 10^4^ cells were seeded in the upper chambers with 200 μL serum-free medium, and 600 μL medium with 20% FBS was added to the lower chambers. Twenty-four hours later, the migrated cells were fixed, stained and photographed.

### Statistical analysis

All analyses were completed using R software (version 4.1.0). Survival differences were compared by the log-rank test in Kaplan–Meier survival analysis. The Wilcoxon test was utilized to evaluate the difference in continuous variables between the two groups. Differences in the distribution of categorical variables between two groups were compared via the chi-square test. Pearson correlation analysis was used to evaluate the correlation between numeric variables. P values were two sided, and a *p* value < 0.05 was considered statistically significant.

## Results

### Construction of a prognostic model in the TCGA cohort

**FIGURE 1:**
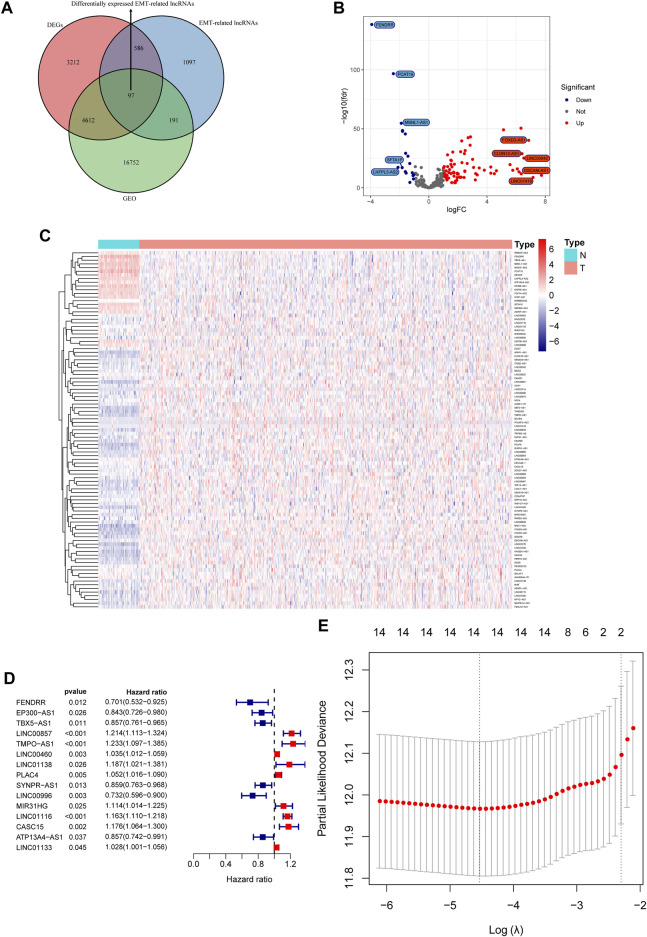
Construction of a prognostic signature based on the expression of EMT-related lncRNAs. **(A)** Venn diagram of differentially expressed EMT-related lncRNAs in GEO and TCGA cohorts. **(B)** Volcano plot of differentially expressed EMT-related lncRNAs. **(C)** Heatmap of differentially expressed EMT-lncRNAs. **(D)** Forest plot of prognostic EMT-related lncRNAs. **(E)** Identification of key prognostic EMT-related lncRNAs via variable selection in LASSO Cox regression.

Differential expression analysis in the TCGA cohort identified a total of 8507 differentially expressed genes (DEGs), among which 97 DEGs were EMT-related lncRNAs that could be found in the GEO datasets (Figure 1A). Ninety-seven differentially expressed EMT-related lncRNAs included 79 lncRNAs that were upregulated in LUAD and 18 lncRNAs that were downregulated in LUAD (Figures 1B,C). Fifteen differentially expressed EMT-related lncRNAs were found to be related to OS in univariate Cox regression analysis (Figure 1D), among which 14 lncRNAs (*FENDRR*, *EP300-AS1*, *LINC00857*, *TMPO-AS1*, *LINC00460*, *LINC01138*, *PLAC4*, *SYNPR-AS1*, *LINC00996*, *MIR31HG*, *LINC01116*, *CASC15*, *ATP13A4-AS1*, *LINC01133*) were selected by LASSO Cox regression analysis to construct a prognostic signature (Figure 1E). Expression of the 14 lncRNAs was displayed in Supplementary Figure S1. The prognostic model was presented as a formula, and the ERPI of all LUAD individuals was calculated based on the expression of 14 prognostic lncRNAs and the corresponding regression coefficients in LASSO Cox regression analysis (Supplementary Table S4).

### Validation of the prognostic model in multiple datasets

**FIGURE 2:**
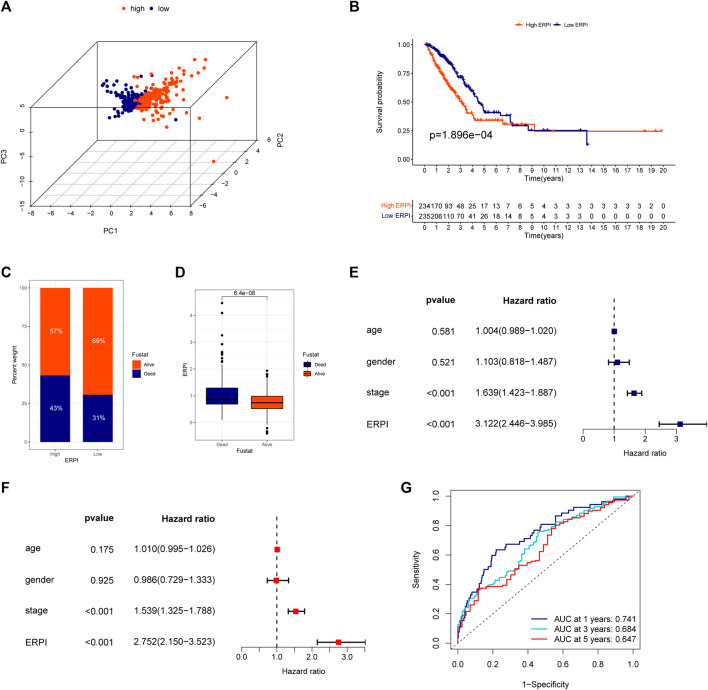
Validation of the ERPI in the TCGA dataset. **(A)** Distribution of high- and low-ERPI groups in PCA based on the expression of 14 EMT-related lncRNAs in the prognostic model in TCGA. **(B)** Survival curves of the high- and low-ERPI groups in TCGA. **(C)** Proportion of dead and living patients in the high- and low-ERPI groups in TCGA. **(D)** ERPI of the dead and living patients. **(E)** Effect of ERPI and clinicopathological parameters on survival in univariate Cox regression analysis in TCGA. **(F)** Effect of ERPI and clinicopathological parameters on survival in multivariate Cox regression analysis in TCGA. **(G)** Time-dependent ROC curves of ERPI in TCGA.

The predictive capacity of ERPI was validated in the TCGA-LUAD dataset which was used as the discovery cohort. The results of PCA demonstrated that the high-ERPI and low-ERPI groups could be separated based on the expression of 14 prognostic EMT-related lncRNAs (Figure 2A). The survival rate of the low-ERPI group was higher than that of the high-ERPI group (*p* < 0.001) (Figure 2B). The proportions of survivors in the high- and low-ERPI groups were 57% and 69%, respectively (Figure 2C). Compared to the survivors, the nonsurviving patients had an elevated ERPI (*p* < 0.001) (Figure 2D). Univariate (HR = 3.122, 95% confidence interval (CI): 2.446–3.985, *p* < 0.001) and multivariate Cox regression analyses (HR = 2.752, 95% CI: 2.150–3.523, *p* < 0.001) revealed that ERPI was an independent prognostic factor affecting survival adversely (Figures 2E,F). Time-dependent ROC curves demonstrated the high sensitivity and specificity of ERPI in predicting survival for patients with LUAD. The AUCs at 1, 3, and 5 years were 0.741, 0.684, and 0.647, respectively (Figure 2G).

**FIGURE 3:**
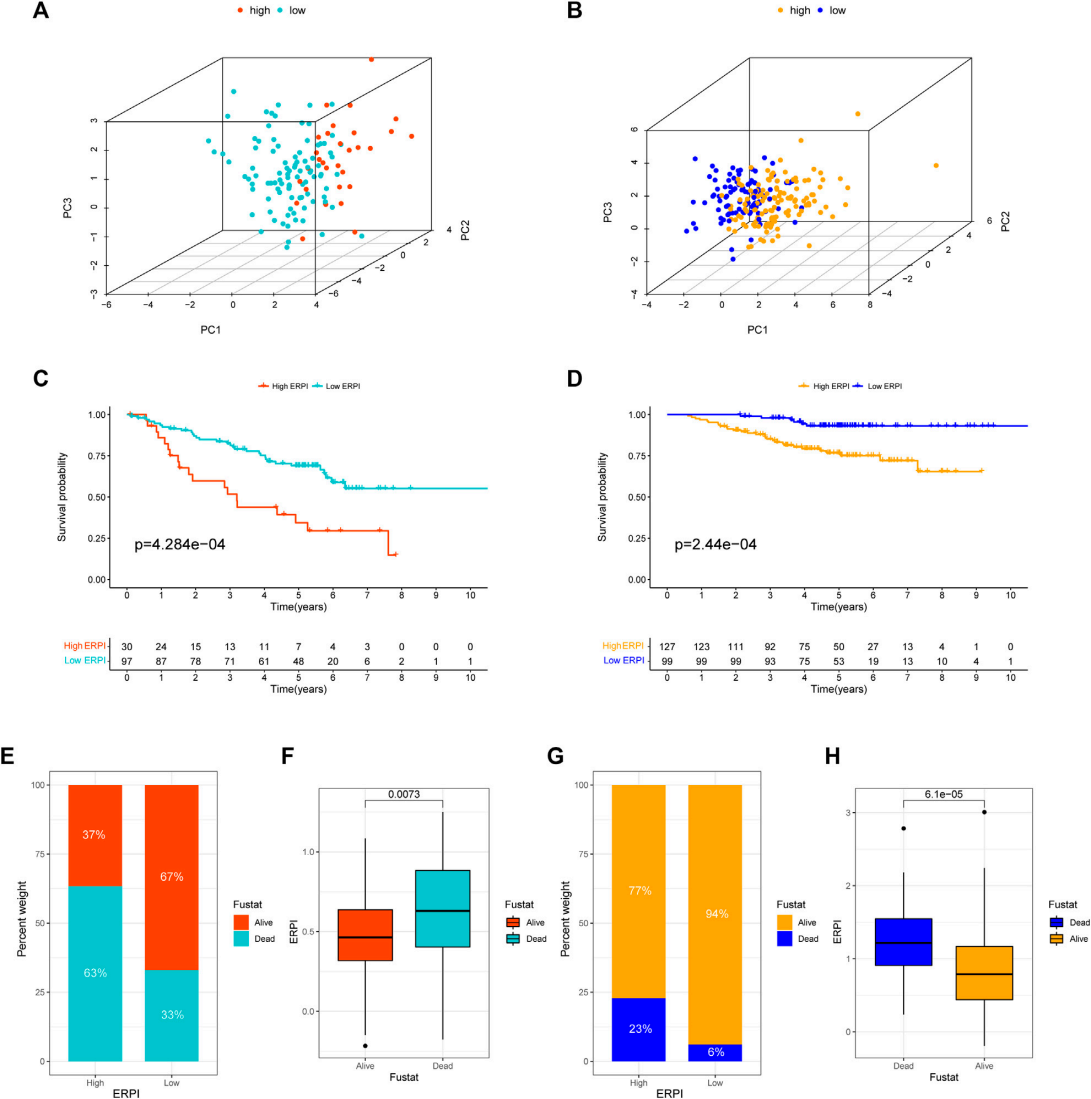
Validation of ERPI in GSE50081 and GSE31210. **(A)** Distribution of high- and low-ERPI groups in PCA based on the expression of 14 EMT-related lncRNAs in the prognostic model in GSE50081. **(B)** Distribution of high- and low-ERPI groups in PCA based on the expression of 14 EMT-related lncRNAs in the prognostic model in GSE31210. **(C)** Survival curves of the high- and low-ERPI groups in GSE50081. **(D)** Survival curves of the high- and low-ERPI groups in GSE31210. **(E)** Proportion of dead and living patients in the high- and low-ERPI groups in GSE50081. **(F)** ERPI of the dead and living patients in GSE50081. **(G)** Proportion of dead and surviving patients in the high- and low-ERPI groups in GSE31210. **(H)** ERPI of the dead and surviving patients in GSE31210.

**FIGURE 4:**
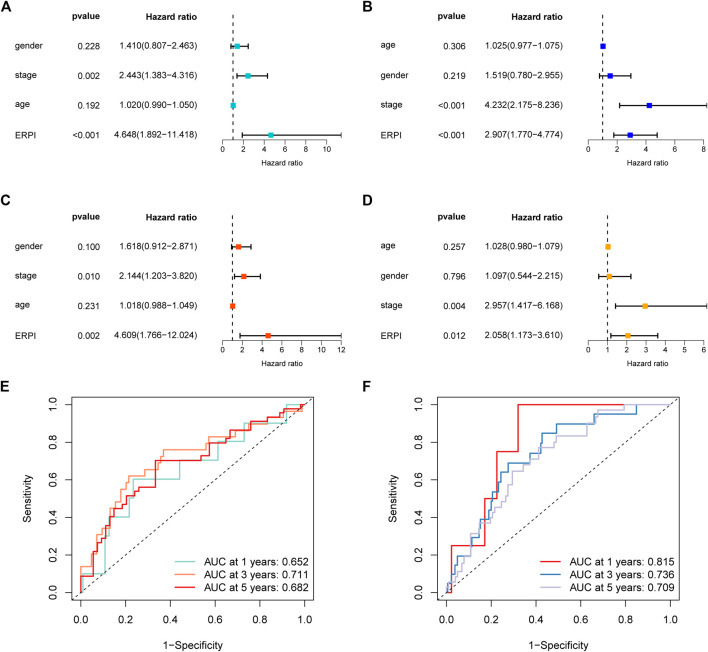
Validation of ERPI in GSE50081 and GSE31210. **(A)** Effect of ERPI and clinicopathological parameters on survival in univariate Cox regression analysis in GSE50081. **(B)** Effect of ERPI and clinicopathological parameters on survival in univariate Cox regression analysis in GSE31210. **(C)** Effect of ERPI and clinicopathological parameters on survival in multivariate Cox regression analysis in GSE50081. **(D)** Effect of ERPI and clinicopathological parameters on survival in multivariate Cox regression analysis in GSE31210. **(E)** Time-dependent ROC curves of ERPI in GSE50081. **(F)** Time-dependent ROC curves of ERPI in GSE31210.

Next, the results of survival analyses in the external cohorts GSE50081 and GSE31210 further confirmed the predictive power of ERPI. The expression of the prognostic EMT-related lncRNAs in the low-ERPI group was significantly different from that in the high-ERPI group in GSE50081 (Figure 3A) and GSE31210 (Figure 3B). The low-ERPI group survived longer than the high-ERPI group in both GSE50081 (*p* < 0.001) (Figure 3C) and GSE31210 (*p* < 0.001) (Figure 3D). In GSE50081, the proportion of survivors in the high- and low-ERPI groups was 37% and 67%, respectively (Figure 3E). In GSE31210, the proportion of survivors in the high- and low-ERPI groups was 77% and 94%, respectively (Figure 3G). The survivors had lower ERPI than the nonsurvivors in both GSE50081 (*p* = 0.0073) (Figure 3F) and GSE31210 (*p* < 0.001) (Figure 3H). ERPI is an indicator of unfavorable survival for patients with LUAD in GSE50081 (HR = 4.648, 95% CI: 1.892–11.418, *p* < 0.001) (Figure 4A) and GSE31210 (HR = 2.907, 95% CI: 1.770–4.774, *p* < 0.001) (Figure 4B). Additionally, the prognostic value of ERPI was not affected by clinical variables in GSE50081 (HR = 4.609, 95% CI: 1.766–12.024, *p* = 0.002) (Figure 4C) and GSE31210 (HR: 2.058, 95% CI: 1.173–3.610, *p* = 0.012) (Figure 4D). The AUCs at 1, 3, and 5 years in GSE50081 were 0.652, 0.711, and 0.682, respectively (Figure 4E). The AUCs at 1, 3, and 5 years in GSE31210 were 0.815, 0.736, and 0.709, respectively (Figure 4F).

**FIGURE 5:**
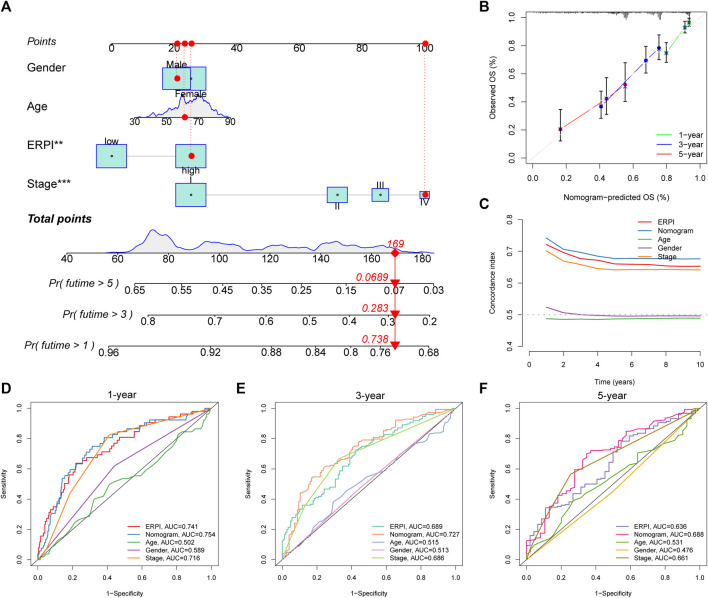
Establishment and validation of the nomogram. **(A)** Nomogram incorporating ERPI and clinicopathological variables. **(B)** Calibration curves of the nomogram. **(C)** C-indexes of the nomogram, ERPI, and clinicopathological variables. **(D)** ROC curves of the nomogram, ERPI, and clinicopathological variables at 1 year. **(E)** ROC curves of the nomogram, ERPI, and clinicopathological variables at 3 years. **(F)** ROC curves of the nomogram, ERPI, and clinicopathological variables at 5 years.

Clinicopathological variables and ERPI are all prognostic factors that can predict survival, so we established a nomogram incorporating ERPI and clinicopathological variables to develop a tool for survival prediction with high predictive accuracy (Figures 5A,B). C-index and ROC curves demonstrated that the nomogram had the best prognostic performance and highest predictive accuracy, followed by ERPI and stage (Figures 5C–F).

### High ERPI was associated with tumor progression

**FIGURE 6:**
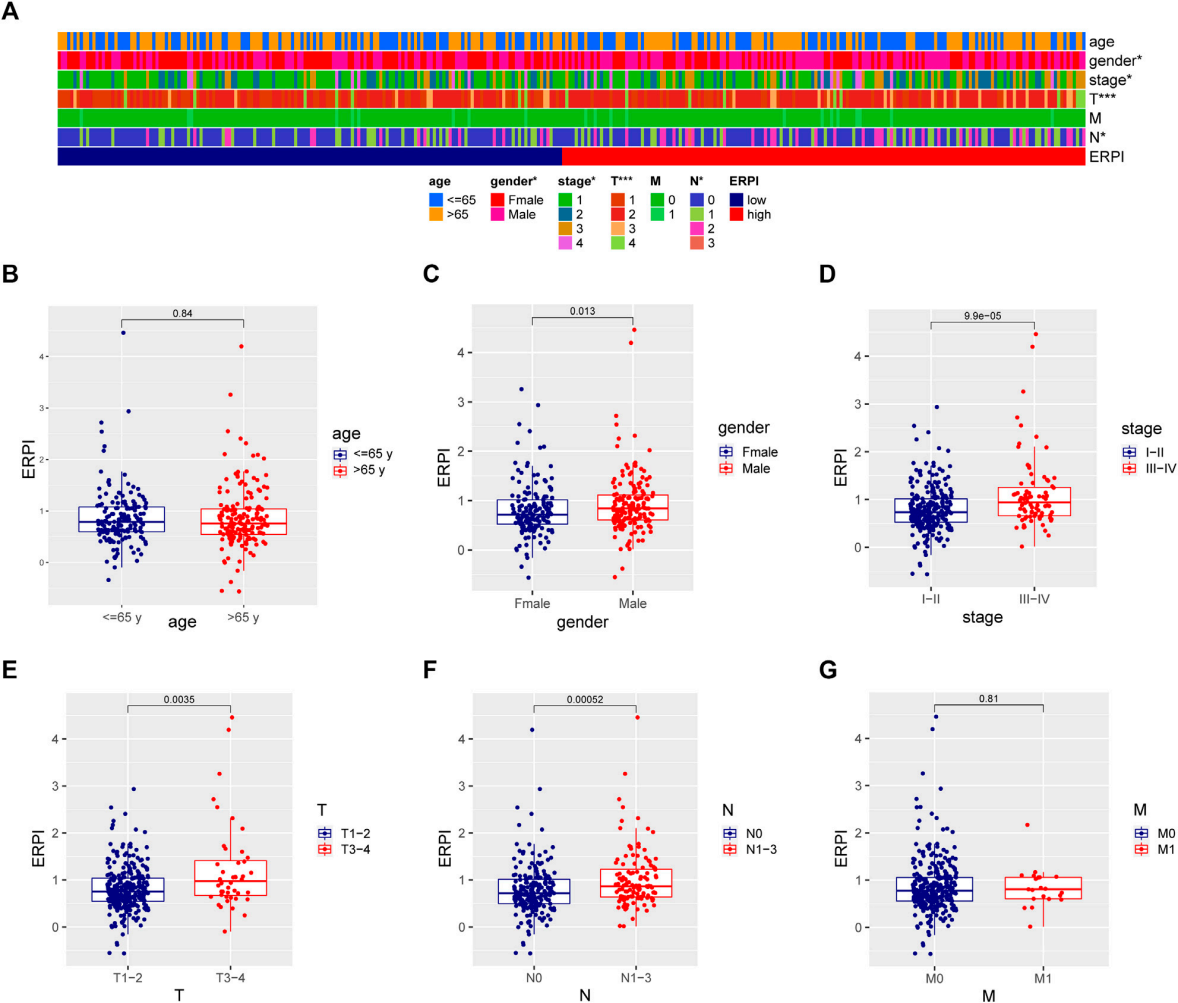
Relationship between ERPI and clinicopathological variables. **(A)** Clinicopathological variables between the high- and low-ERPI groups. **(B–G)** ERPI between subgroups divided by clinicopathological variables.

Compared to the low-ERPI group, the high-ERPI group had an elevated proportion of male patients and patients with large primary tumors or lymph node metastasis (Figure 6A). Most of the patients in advanced stage were distributed in the high-ERPI group, indicating that ERPI was related to malignant progression. Meanwhile, when the patients were grouped according to the clinicopathological variables, increased ERPI was observed in male patients and patients in an advanced stage (Figures 6B–F). Although ERPI was correlated with large primary tumors and lymph node metastasis, no association was observed between ERPI and distant metastasis (Figures 6A,G).

### The high- and low-ERPI groups had different TME characteristics

**FIGURE 7:**
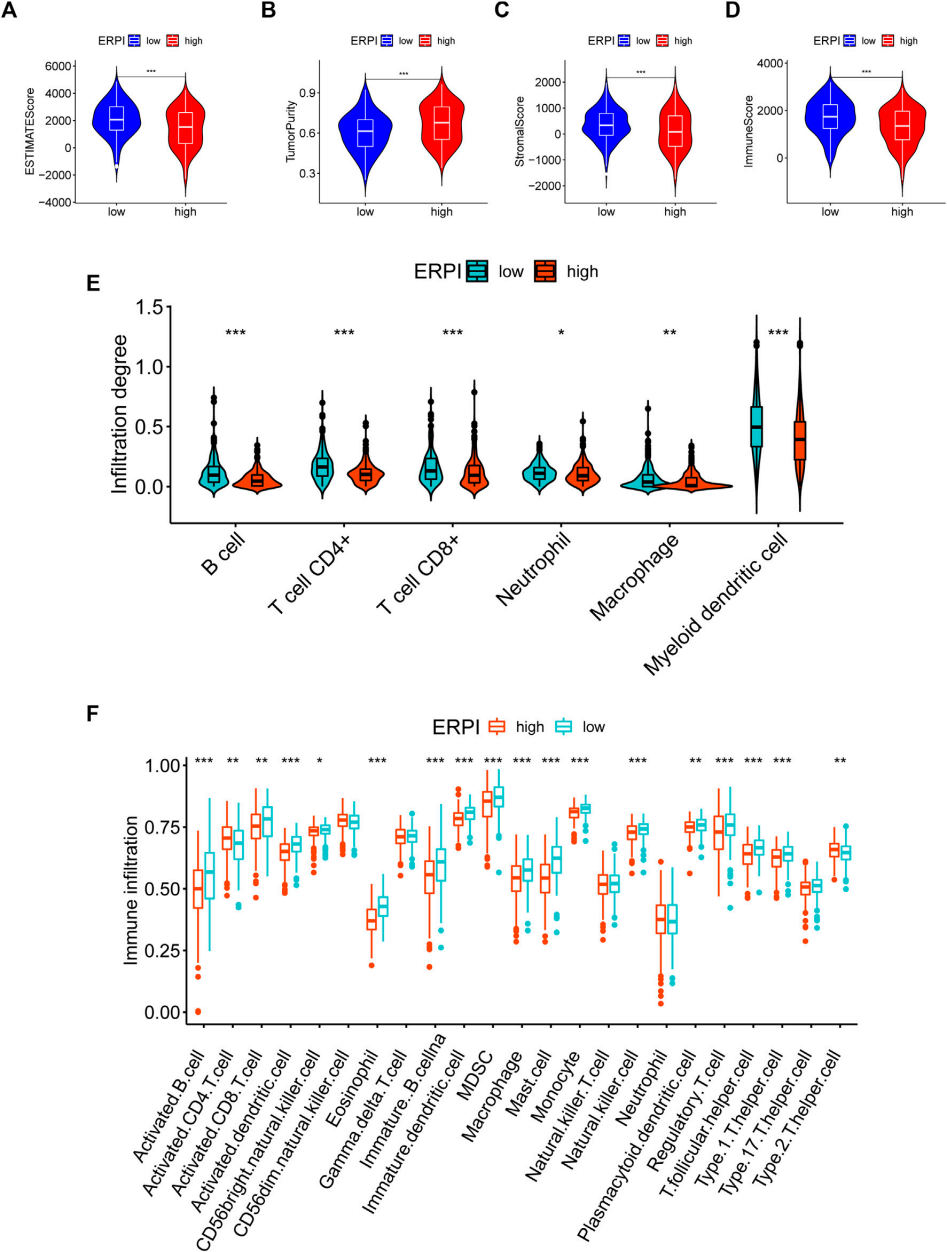
TME characteristics in the high- and low-ERPI groups. **(A–D)** ESTIMATE score, tumor purity, stromal score, and immune score between the high- and low-ERPI groups. **(E)** Abundance of immune cell subsets acquired via TIMER between the high- and low-ERPI groups. **(F)** Abundance of immune cell subsets acquired via ssGSEA between the high- and low-ERPI groups.

**FIGURE 8:**
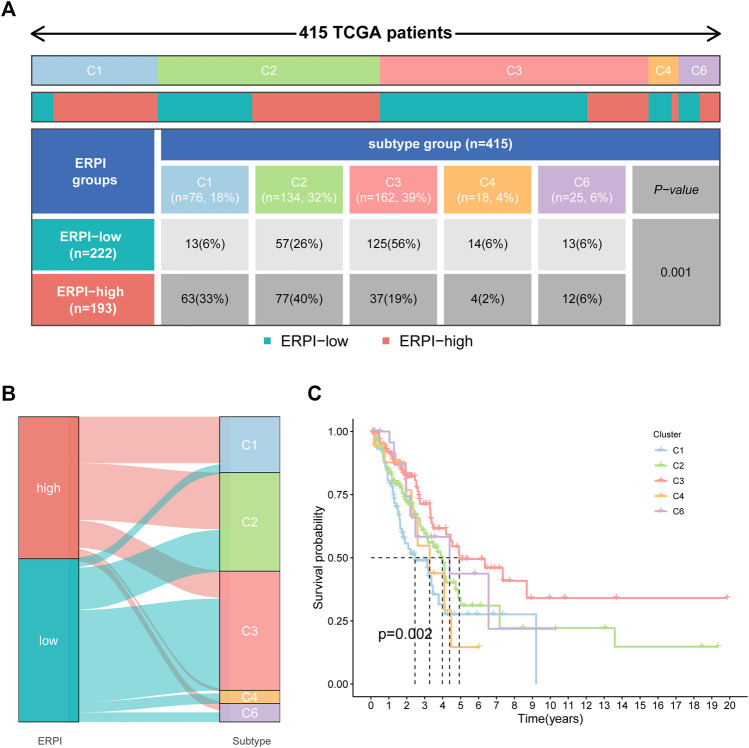
Correlation between ERPI and immune subgroups. **(A)** Percentage of immune subgroups in the high- and low-ERPI groups. **(B)** Distribution of immune subgroups in the high- and low-ERPI groups. **(C)** Survival curves of the immune subgroups.

Compared to the high-ERPI group, the low-ERPI group had a significantly higher proportion of stromal cells and immune cells and a lower proportion of tumor cells in the TME (Figures 7A–D). Immune cells are fundamental components of the TME and can be classified into diverse subtypes that have different roles in tumor progression and antitumor immunity. We analysed the relationship between immune cell subsets and the ERPI via the TIMER and ssGSEA algorithms. The low-ERPI group had not only higher abundance of CD8^+^ T cells, CD4^+^ T cells, B cells, and dendritic cells (DCs) but also higher infiltration degree of activated CD8^+^ T cells, B cells, and DCs than the high-ERPI group (Figures 7E,F). These results revealed that the infiltration and function of immune cells in the TME were suppressed in the high-ERPI group. Then, we analysed the immune subtypes identified in another study to further elucidate the relationship between ERPI and tumor immunity. C3 accounted for the majority of the low-ERPI group, whereas C1 and C2 were the most common immune subtypes in the high-ERPI group (Figures 8A,B). The survival rate of C3 was the highest among all the immune subtypes (Figure 8C).

### Functional annotation of ERPI groups

**FIGURE 9:**
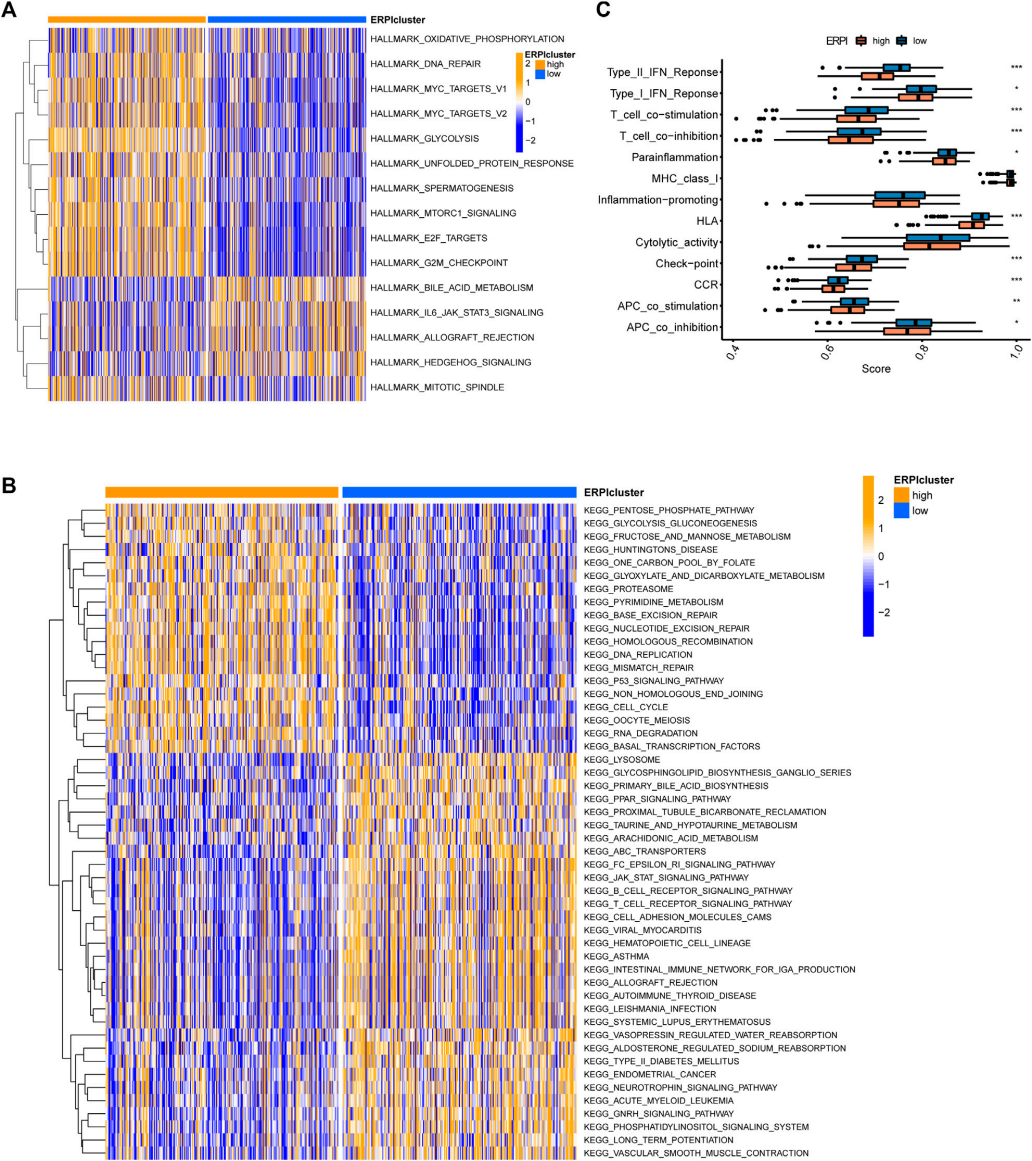
Functional annotation of the ERPI groups. **(A)** Differences in hallmarks between the high- and low-ERPI groups in GSVA. **(B)** Differences in pathways between the high- and low-ERPI groups in GSVA. **(C)** Differences in immune processes between the high- and low-ERPI groups in ssGSEA.

GSVA in hallmarks revealed that the high-ERPI group was associated with the activation of hallmarks such as oxidative phosphorylation, DNA repair, MYC targets, glycolysis, E2F targets, and mTORC1 signaling (Figure 9A). Regarding pathways, DNA replication, p53 signaling pathway, and mismatch repair were upregulated in the high-ERPI group, while the B-cell and T-cell receptor signaling pathways and pathways related to cell adhesion were enriched in the low-ERPI group (Figure 9B). The ssGSEA results indicated that immune processes, including the interferon response, parainflammation, chemokine receptor, T-cell co-inhibition, human leucocyte antigen, and antigen presenting cell co-inhibition, were activated in the low-ERPI group (Figure 9C). The ssGSEA results suggested that the low-ERPI group had a stronger immune response than the low-ERPI group.

### LINC01138 promotes progression of lung cancer cells

**FIGURE 10:**
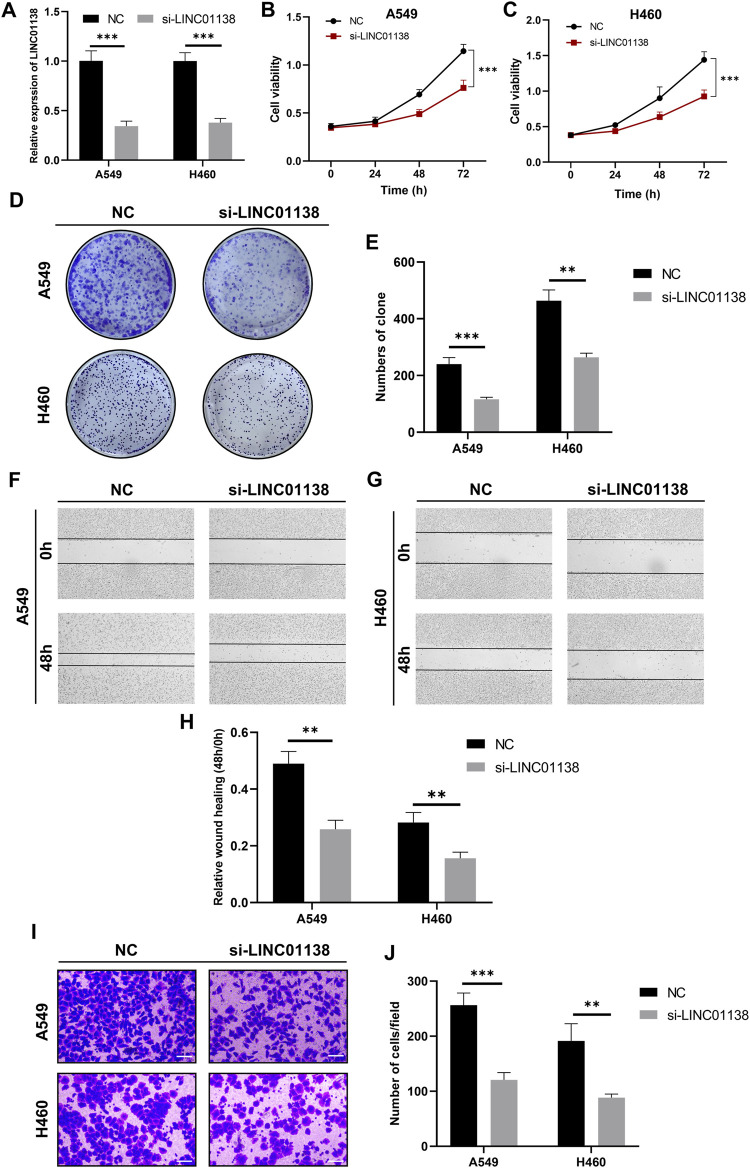
Knockdown of LINC01138 repressed viability, proliferation, and migration of A549 and H460 cells. **(A)** Expression of LINC01138 in A549 and H460 cells after its knockdown. **(B,C)** CCK-8 assay results showing viability of A549 and H460 cells after LINC01138 knockdown. **(D,E)** The proliferation of A549 and H460 cells evaluated using colony formation assay. **(F–H)** Migration capacity of A549 and H460 cells tested using wound-healing assay. **(I,J)** Migration capacity of A549 and H460 cells tested using Transwell assay. The scale bars represent 200 μm.

Among these key signature lncRNAs, LINC01138 was reported to promote tumor progression in liver cancer, gastric cancer, glioma and kidney malignancy; however, whether *LINC01138* promotes lung cancer progression remains unclear. Therefore, we investigated the effects of *LINC01138* on malignant behaviors of lung cancer cells through transfection with its specific siRNA, and the interfering efficiency was assessed by RT-qPCR (Figure 10A). Then, we checked the effect of *LINC01138* on the proliferation of lung cancer cells through CCK8 and clone formation assays. The results of the CCK8 assay showed that silencing LINC01138 dramatically decreased the viability of lung cancer cells (*p* < 0.001) (Figures 10B,C). In addition, the clone formation assay also indicated that silencing *LINC01138* significantly suppressed clone formation of lung cancer cells (*p* < 0.01) (Figures 10D,E). These results suggested that silencing *LINC01138* could inhibit lung cancer cells’ proliferation. Next, we further explored the role of LINC01138 in tumor metastasis through wound healing and transwell assays. The results showed that silencing *LINC01138* markedly reduced wound healing ability of lung cancer cells (*p* < 0.01) (Figures 10F–H) as well as the migration capability (*p* < 0.01) (Figures 10I,J). The results demonstrated that silencing *LINC01138* represses lung cancer metastasis *in vitro*. Altogether, the above results suggested that *LINC01138* accelerates progression of lung cancer cells.

## Discussion

The 5-year survival rate of patients with lung cancer is 22%, which is as low as 6% in the case of distant metastasis (Siegel et al., 2022). Lung cancer consists of divergent histological subtypes, among which LUAD is the most frequently diagnosed subtype. Powerful tools for survival prediction are necessary to make precise clinical decisions and individual treatment strategies for patients with LUAD. EMT is a critical cellular program in malignant progression, and EMT-related lncRNAs have been proven to regulate the development of cancer. Nevertheless, few studies have explored the potential of EMT-related lncRNAs in survival prediction in LUAD.

To explore novel survival predictors, we analysed TCGA-LUAD data to identify prognostic EMT-related lncRNAs. A prognostic signature was constructed based on the expression of 14 EMT-related lncRNAs (*FENDRR*, *EP300-AS1*, *LINC00857*, *TMPO-AS1*, *LINC00460*, *LINC01138*, *PLAC4*, *SYNPR-AS1*, *LINC00996*, *MIR31HG*, *LINC01116*, *CASC15*, *ATP13A4-AS1*, and *LINC01133*). *FENDRR, EP300-AS1, SYNPR-AS1*, *LINC00996*, and *ATP13A4-AS1* are tumor suppressor genes whose overexpression is associated with favorable outcome, whereas the other genes in the model are oncogenes. The results of the studies exploring the functions of prognostic EMT-related lncRNAs in cancers were consistent with our study. *FENDRR* was reported to be positively correlated with survival in prostate cancer and renal cell carcinoma (He et al., 2019; Liu et al., 2019). *FENDRR* has also been demonstrated to impair the invasion capacity of non-small cell lung cancer (NSCLC) and genitourinary system malignancies (Zhang et al., 2019a; Zhang et al., 2019b; Zhu et al., 2020). One study suggested that knockdown of *LINC00857* suppressed the viability of bladder cancer cells and sensitized bladder cancer cells to cisplatin (Dudek et al., 2018). Silencing *LINC00857* inhibited the malignant behaviors of colorectal cancer and pancreatic cancer (Chang et al., 2021; Li et al., 2021; Meng et al., 2021). Xia C et al. reported that *LINC00857* enhanced EMT to promote the invasion capacity of hepatocellular carcinoma (Xia et al., 2018). *LINC00460* acts as an oncogene in breast cancer and gastric cancer, and its high expression is correlated with unfavorable outcomes in patients with breast cancer (Zhang et al., 2019c; Zhu et al., 2019). A number of experimental studies revealed that *LINC00460* promoted invasion and metastasis in various malignancies. *LINC00460* was reported to promote the viability and migration of NSCLC (Zhao et al., 2019). Zhang J et al. observed that *LINC00460* controls radiation sensitivity of colon cancer by modulating EMT (Zhang et al., 2020). Another study suggested that *LINC00460* promoted EMT and metastatic potential of esophageal cancer (Cui et al., 2020). The role of *LINC00460* as an oncogene was also reported in hepatocellular carcinoma, pancreatic cancer, kidney malignancy, and prostate cancer (Dong and Quan, 2019; Yang et al., 2020b; Cheng et al., 2021). Overexpression of *MIR31HG* was associated with malignant progression and high infiltration degree of immune cells in thyroid cancer (Chen et al., 2022). A meta-analysis suggested that overexpression of *MIR31HG* predicted poor survival and metastasis in respiratory system and digestive system tumors (Wei et al., 2022). *MIR31HG* acted as a predictor of survival and risk of recurrence for patients with colorectal cancer (Zhang et al., 2019d; Eide et al., 2019). Additionally, increased expression of *MIR31HG* has been demonstrated to promote tumor progression and reduce the efficacy of gefitinib in NSCLC (Wang et al., 2017; Ning et al., 2018; Dandan et al., 2019). *MIR31HG* also exhibited oncogenic properties in breast cancer and esophageal squamous cell carcinoma (Sun et al., 2018; Xin et al., 2021).

Studies have demonstrated that *LINC01138* promotes malignant behavior in liver cancer, gastric cancer, glioma, and kidney malignancy (Li et al., 2018; Zhang et al., 2018; Dou et al., 2019; Xu et al., 2021). However, no mechanistic exploration has been made to clarify the role of *LINC01138* in LUAD. In this study, we found that *LINC01138* was upregulated in LUAD and that upregulation of *LINC01138* was associated with poor survival by analyzing expression data in TCGA. Then, we conducted *in vitro* experiments to further clarify the exact role of *LINC01138* in the progression of lung cancer. Silencing LINC01138 repressed viability, proliferation, and metastasis of A549 and H460 cells, indicating that *LINC01138* was an oncogene in lung cancer. Our findings and other studies suggest that the oncogenic properties of *LINC01138* may be universal across cancers.

Each patient could obtain a score named ERPI, which served as a classifier to distinguish patients with favorable prognosis from those with poor prognosis. ERPI is a reliable survival predictor for patients with LUAD, and the predictive capacity of ERPI is independent of clinicopathological factors. High ERPI is associated with advanced TNM stage, which also predicts poor survival for patients. Given that clinicopathological variables are also robust prognostic factors, we established a nomogram incorporating ERPI and clinicopathological variables to further improve the prognostic performance of these factors. The C-index and ROC curves demonstrated that the nomogram improved the prognostic performance of the ERPI and TNM stage. The nomogram can become a simple tool with high predictive accuracy for survival prediction in clinical practice. GSVA revealed the overactivation of mTORC1 signaling and MYC targets in the high-ERPI group. Dysregulation of mTOR signaling is associated with the initiation and progression of cancer, and overactivated mTORC1 signaling has been demonstrated to promote malignant behaviors of cancer cells (Kim et al., 2017). *MYC* is an oncogene supporting oncogenic processes and resistance to therapy (Massó-Vallés et al., 2020). *MYC* deregulation has been proven to accelerate oncogenesis and program stroma to induce immune suppression in lung cancer (Kortlever et al., 2017). These findings indicated that oncogenic processes are overactivated in the high-ERPI group.

Recruitment of immune cells and activation of immune processes are associated with not only tumor progression but also the efficacy of immune checkpoint inhibitors (ICIs), which are effective treatments for a subset of LUAD individuals (Quail and Joyce, 2013; Thommen et al., 2018; Helmink et al., 2020; Bagchi et al., 2021). Studies have suggested that EMT dampens the functions of immune cells, induces immune evasion, and promotes resistance to immunotherapy (Kudo-Saito et al., 2009; Akalay et al., 2013a; Akalay et al., 2013b). Mesenchymal carcinoma cells display increased resistance to immune attack and induce formation of immunosuppressive cells (Dongre et al., 2017). We analyzed the relationship between the ERPI and TME characteristics. The low-ERPI group had a higher percentage of immune cells and stromal cells and lower tumor purity than the high-ERPI group. ERPI was negatively related to the abundance of immune cells and activated immune cells, including CD8^+^ cells, B cells, and DCs. The abundance and function of these immune cell subsets are associated with survival outcome and response to ICIs. For example, single-cell sequencing analyses suggested that exhaustion of T cells correlated with survival outcome in LUAD individuals (Guo et al., 2018). One study identified a CD8^+^ T-cell subset whose high abundance was proven to be related to resistance to ICIs (Sanmamed et al., 2021). DCs are critical mediators of antigen presentation, promoting T-cell activity and immune control (Böttcher et al., 2018; Ahluwalia et al., 2021). One study suggested that DC deficiency led to dysfunction in immune surveillance (Hegde et al., 2020). DCs were demonstrated to be crucial targets of ICI treatment, dictating the efficacy of PD-L1 blockade (Mayoux et al., 2020). NSCLC individuals treated with atezolizumab with a high DC signature had improved OS compared with those with a low DC signature (Mayoux et al., 2020). High abundance of B cells was also reported to indicate favorable prognosis in patients with LUAD (Cui et al., 2021). Immune-related responses, including the IFN response and HLA, were enhanced in the low-ERPI group and are critical processes in antitumor immunity. Impaired HLA class I antigen processing reduces responsiveness to ICIs in lung cancer (Gettinger et al., 2017). Type I IFN responses can activate the immune system and repress tumor progression (Gozgit et al., 2021). IFN enhances the expression of immunomodulatory molecules to augment the activities of CD8^+^ T cells and NK cells (Edwards et al., 1985; Hervas-Stubbs et al., 2012; Zitvogel et al., 2015). IFN can also induce the formation of antitumorigenic cells and repress the accumulation of immunosuppressive cells (Duluc et al., 2009; Sisirak et al., 2012). High abundance of immune cells and highly activated immune processes may be one of the reasons why the low-ERPI group showed survival advantage over the high-ERPI group.

The present study has several limitations. The prognostic signature was constructed and tested retrospectively based on LUAD cohorts in public databases. A large and prospective LUAD cohort is necessary to validate the prognostic performance of ERPI and evaluate whether clinical application of ERPI can help patients acquire survival benefits. ICIs are effective treatments for a subset of patients with LUAD. Although the high- and low-ERPI groups had different characteristics of immune infiltration and immune response, the relationship between ERPI and efficacy of ICIs in LUAD was not explored due to lack of LUAD cohorts treated with ICIs. In addition, only the function of *LINC01138* was clarified in lung cancer, whereas the mechanisms underlying other prognostic EMT-related lncRNAs have not been elucidated.

In conclusion, this study explored the prognostic EMT-related lncRNAs in LUAD to identify a prognostic signature that served as a classifier to predict outcome for patients with LUAD. We also constructed a nomogram that could be used as a simple tool for survival prediction. Additionally, our study clarified the biological function of LINC01138 in tumorigenesis, indicating that it could promote malignant behaviors in lung cancer. Our study lays foundation for stratification of LUAD individuals to achieve personalized treatment and contributes to understanding the role of EMT-related lncRNAs in LUAD.

## Data Availability

The original contributions presented in the study are included in the article/Supplementary Material, further inquiries can be directed to the corresponding authors.

## Abbreviations

EMT, epithelial-mesenchymal transition; lncRNAs, long noncoding RNAs; LUAD, lung adenocarcinoma; TCGA, The Cancer Genome Atlas; LASSO, Least Absolute Shrinkage and Selection Operator; ERPI, EMT-related prognostic index; TME, tumor microenvironment; ESTIMATE, Estimation of STromal and Immune cells in Malignant Tumours using Expression data; TIMER, Tumor Immune Estimation Resource; ssGSEA, single sample gene set enrichment analysis; GSVA, Gene set variation analysis; OS, overall survival; GEO, Gene Expression Omnibus; DEGs, differentially expressed genes; FDR, false discovery rate; FC, fold change; ROC, receiver operating characteristic; AUC, area under the curve; CI, confidence interval; DC, dendritic cell; NSCLC, non-small cell lung cancer; ICIs, immune checkpoint inhibitors.
