# Supplementary material for: Identification of a prognostic classifier based on EMT-related lncRNAs and the function of LINC01138 in tumor progression for lung adenocarcinoma
Source: Front Mol Biosci. 2022 Aug 17;9:976878. doi: 10.3389/fmolb.2022.976878 (PMC9428519; doi:10.3389/fmolb.2022.976878)
Supplement: Supplementary file 3 [file Table1.DOCX]

Table S1 Clinical information of the LUAD cohorts.

| Parameters | Total | TCGA | GSE50081 | GSE31210 |
| --- | --- | --- | --- | --- |
| Age (years) |  |  |  |  |
| <=65 | 441(53.65%) | 225(47.97%) | 40(31.5%) | 176(77.88%) |
| >65 | 381(46.35%) | 244(52.03%) | 87(68.5%) | 50(22.12%) |
| Gender |  |  |  |  |
| Female | 435(52.92%) | 252(53.73%) | 62(48.82%) | 121(53.54%) |
| Male | 387(47.08%) | 217(46.27%) | 65(51.18%) | 105(46.46%) |
| Stage |  |  |  |  |
| I | 514(62.53%) | 254(54.16%) | 92(72.44%) | 168(74.34%) |
| II | 206(25.06%) | 113(24.09%) | 35(27.56%) | 58(25.66%) |
| III | 77(9.37%) | 77(16.42%) | 0(0%) | 0(0%) |
| IV | 25(3.04%) | 25(5.33%) | 0(0%) | 0(0%) |
| Survival status |  |  |  |  |
| Alive | 563(68.49%) | 296(63.11%) | 76(59.84%) | 191(84.51%) |
| Dead | 259(31.51%) | 173(36.89%) | 51(40.16%) | 35(15.49%) |
