# Supplementary figures and images for: Identification of a prognostic classifier based on EMT-related lncRNAs and the function of LINC01138 in tumor progression for lung adenocarcinoma
Source: Front Mol Biosci. 2022 Aug 17;9:976878. doi: 10.3389/fmolb.2022.976878 (PMC9428519; doi:10.3389/fmolb.2022.976878)

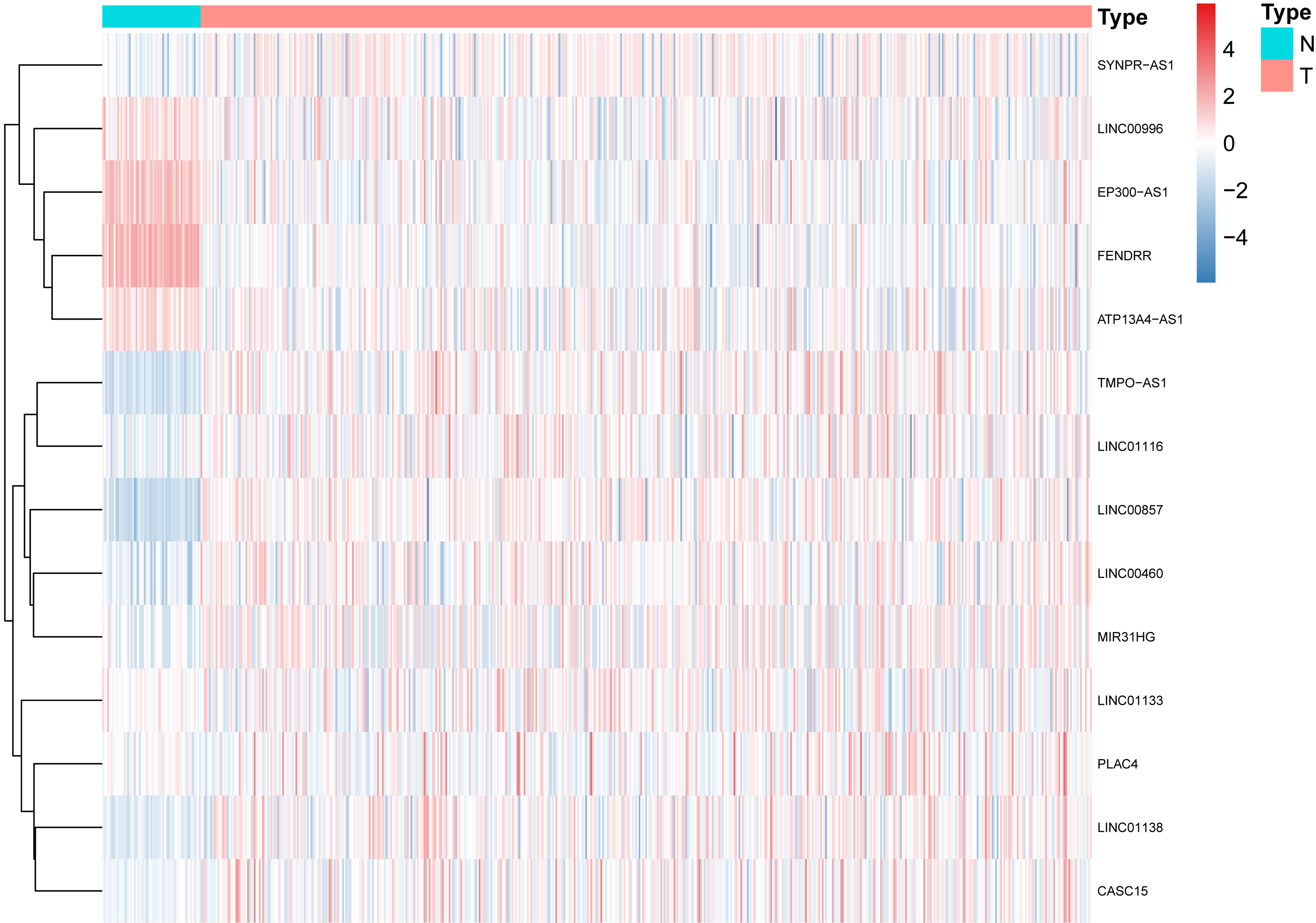

Supplement: Supplementary file 4 [file Image1.TIF]
