# Supplementary material for: Identification of a prognostic classifier based on EMT-related lncRNAs and the function of LINC01138 in tumor progression for lung adenocarcinoma
Source: Front Mol Biosci. 2022 Aug 17;9:976878. doi: 10.3389/fmolb.2022.976878 (PMC9428519; doi:10.3389/fmolb.2022.976878)
Supplement: Supplementary file 5 [file Table4.DOCX]

Table S4 LncRNAs and their coefficients of the prognostic model.

| Gene | Coef |
| --- | --- |
| FENDRR | -0.06270885 |
| EP300.AS1 | -0.034232299 |
| LINC00857 | 0.114664051 |
| TMPO.AS1 | 0.040395579 |
| LINC00460 | 0.028118229 |
| LINC01138 | 0.086949357 |
| PLAC4 | 0.039088142 |
| SYNPR.AS1 | -0.059809847 |
| LINC00996 | -0.148983484 |
| MIR31HG | 0.008284901 |
| LINC01116 | 0.1175697 |
| CASC15 | 0.067787325 |
| ATP13A4.AS1 | -0.022463759 |
| LINC01133 | 0.021991296 |
